# Repeated hands-and-knees positioning during labour: a randomized pilot study

**DOI:** 10.7717/peerj.25

**Published:** 2013-02-12

**Authors:** Ellen D. Hodnett, Robyn Stremler, Stephen H. Halpern, Julie Weston, Rory Windrim

**Affiliations:** 1 Lawrence S. Bloomberg Faculty of Nursing, University of Toronto, Toronto, Canada; 2 Research Institute, The Hospital for Sick Children, Toronto, Canada; 3 Department of Anaesthesia, Faculty of Medicine, University of Toronto, Toronto, Canada; 4 Department of Anaesthesia, Sunnybrook Health Sciences Centre, Toronto, Canada; 5 Department of Obstetrics and Gynaecology, Mount Sinai Hospital, Toronto, Canada; 6 Department of Obstetrics and Gynaecology, Faculty of Medicine, University of Toronto, Canada

**Keywords:** Randomized controlled trial, Spontaneous delivery, Persistent back pain, Pilot study, Labour, Hands-and-knees position

## Abstract

**Background.** Caesarean birth rates in North America continue to rise, in the absence of benefit for mothers and babies. One reason may be that hospitalized labouring women spend most of their labours in recumbent or semi-recumbent positions. Although hands-and-knees position has theoretical advantages, efforts to encourage its adoption in practice are severely hampered by the lack of compelling evidence that it is beneficial. Before a definitive, large scale trial, with spontaneous vaginal birth as the primary outcome, could be justified in terms of time, effort, and expense, several feasibility and acceptability questions had to be addressed. We aimed to enrol 60 women in a pilot study to assess feasibility and acceptability of the trial protocol, and to obtain estimates of treatment effects on method of birth and persistent back pain.

**Methods.** We conducted a pilot study at two North American hospitals. In ten months of recruitment, 30 nulliparous women in labour at term were randomly allocated to either usual care (use of any position during labour except hands-and-knees) or to try hands-and-knees for 15 min every hour during labour. Data were collected about compliance, acceptability, persistent back pain, intrapartum interventions, and women’s views of their experiences.

**Results.** Although mean length of time from randomization to delivery was over 12 hours, only 9 of the 16 women allocated to repeated hands-and-knees used it more than twice. Two of the 14 in the usual care group used hands-and-knees once. Twenty-seven women had regional analgesia (15 in the hands-and-knees group and 12 in the usual care group). Eleven in the hands-and-knees group and 14 in the usual care group had spontaneous vaginal births. One woman (in the hands-and-knees group) had a vacuum extraction. Four women in the hands-and-knees group and none in the usual care group gave birth by caesarean section. Hourly back pain ratings were highly variable in both groups, covering the full range of possible scores. Given the low compliance with the hands-and-knees position, it was not possible to explore relationships between use of the position and persistent back pain scores. When asked to rate their overall satisfaction with their birth experiences, the hands-and-knees group’s ratings tended to be lower than those in the usual care group, although 11 in the hands-and-knees group and 8 in the usual care group stated they would probably or definitely try the position in a subsequent labour.

**Conclusion.** We concluded that we could not justify the time and expense associated with a definitive trial. However such a trial could be feasible with modifications to eligibility criteria and careful selection of suitable settings.

## Introduction

Despite widespread and varied efforts to reduce Caesarean delivery rates during the past two decades, rates continue to rise. In 2009 in the 34 countries in the Organization for Economic Co-operation and Development, the mean rate was 26% (OECD, 2012). Caesarean delivery carries a two-to-threefold risk of maternal mortality (Villar et al., 2007; Deneux-Tharaux et al., 2006) and an increased risk of maternal and perinatal morbidity (Villar et al., 2007). There is a need for simple, low cost interventions to increase the likelihood of normal vaginal birth.

Two large North American trials and two national USA surveys found that labouring women in North American hospitals have high rates of medical intervention; between 77%–94% of women had continuous electronic fetal heart rate monitoring, 63%–85% had regional analgesia, and 62%–84% had intravenous oxytocin (Declercq et al., 2002; Declercq et al., 2006; Hodnett et al., 2002; Hodnett et al., 2008). Given the restrictions on movement posed by these interventions, it is not surprising that hospitalized women in labour spend nearly all of their time in passive positions, such as semi-sitting, sitting, semi-recumbent, and side-lying. Recumbent positions (supine or semi-sitting) can result in poor alignment of the presenting part with the pelvic canal (Andrews & Andrews, 2004). However, while non-recumbent positions have theoretical advantages, there is scant good quality evidence of effectiveness (Hunter, Hofmeyr & Kulier, 2007). The labour position with the best evidence of benefit is hands-and-knees (Stremler et al., 2005). Hands-and-knees position involves the labouring woman “on all fours”, i.e. like a baby who is crawling, so that her abdomen is suspended and her hips are at right angles to the floor or bed. The position can be assumed by a woman without leaving her labour bed, by women who have had low dose regional analgesia, and by those who are connected to electronic fetal monitors and intravenous lines.

Mechanisms whereby hands-and-knees position during labour can increase the likelihood of spontaneous vaginal birth include pain/stress reduction, improved uterine blood flow, and enhanced fetopelvic relationships (Stremler et al., 2005; Andrews & Andrews, 2004; Biancuzzo, 1993; Fenwick & Simkin, 1987). Our first pilot trial of hands-and-knees position (LPT1, *n* = 147) provided evidence of benefit for women labouring with a fetus in the occipitoposterior (OP) position (Stremler et al., 2005). Women randomized to the experimental group were in hands-and-knees position for a minimum of 30 min within a one-hour intervention period. LPT1 showed that hands-and-knees reduced persistent back pain, it was acceptable to labouring women, and it is a position that can be assumed in bed, in women with low dose epidural analgesia, continuous electronic fetal heart rate monitoring, and intravenous infusions. The pilot trial demonstrated that even a short intervention was highly suggestive of benefit in rotating from OP to occipitoanterior (OA) position (RR = 2.4, 95% confidence interval 0.88, 6.62, number needed to treat = 11).

LPT1 involved only labouring women with a fetus in the OP position, but fetal position is an unstable phenomenon during labour, and the majority of OP positions at birth are not OP earlier in labour (Peregrine, O’Brien & Jauniaux, 2007; Lieberman et al., 2005; Melzack, Belanger & Lacroix, 1991; Simkin, 1995). An important clinical question is whether hands-and-knees position should be recommended to all low-risk women during labour, regardless of fetal head position at trial entry. If so, the position would need to be tried repeatedly during labour, since fetal position is unstable.

Before a definitive, large scale trial, with spontaneous vaginal birth as the primary outcome, could be justified in terms of time, effort, and expense, several feasibility and acceptability questions had to be addressed. The objectives of our second pilot trial (“LPT2”) were: 1) to provide an estimate of enrolment rates; 2) to assess compliance with the study protocol by participants and care providers; 3) to obtain women’s views about their experiences using the hands-and-knees position; and 4) to provide estimates of treatment effects to inform the sample size calculation for a large trial. The protocol was registered at www.clinicaltrials.gov, registration number NCT01720004.

## Materials and Methods

The settings for LPT2 were two North American hospitals, one in Toronto, Canada and one in Fort Worth, Texas. The study was approved by the research ethics boards at the University and the participating hospitals. Prior to beginning enrolment at a trial site, the principal investigator and trial coordinator gave presentations to the obstetrical and nursing staff, to ensure everyone understood the protocol. The hospital research nurse followed up with training sessions with the nursing staff, to ensure that all nurses: 1) understood trial procedures (in particular that women in the usual care group were not to be offered hands-and-knees position, and women in the hands-and-knees group were to be encouraged to try hands-and-knees repeatedly during labour); 2) were comfortable with assisting women into hands-and-knees position; and 3) understood the trial enrolment procedure, including accessing the trial website for randomization.

The goal was to enrol 60 women, at hospitals in which the regional analgesia used for labour was sufficiently low dose that women could move their legs and safely assume the hands-and-knees position. Eligible women were: nulliparous; ≥37 weeks gestation; in established early labour; anticipating a vaginal delivery of a single fetus in the cephalic position; and competent to give informed consent.

Women were excluded if delivery was anticipated within 3 h (and thus there would be little opportunity for repeated hands and knees positioning), if they had a medical contraindication or physical limitation such that hands-and-knees position was contraindicated, or if they had a doula or midwife who encouraged the use of hands-and-knees position.

Eligible women were identified, informed about the study, and informed consent was obtained from those who were willing to participate. If the woman did not have regional (i.e. epidural or spinal-epidural analgesia) in situ, she was asked if she thought she would want one within the next hour, and if so, enrolment was delayed until after the regional analgesia had been administered. With a 1:1 allocation ratio, randomization was centrally controlled and concealed, using www.randomize.net. After collection of baseline data, the nurse accessed the study website to obtain the woman’s group assignment.

### The interventions

#### Usual care

Most of labour occurs in bed, usually in semi-recumbent, sitting, or side-lying positions, although women were permitted/encouraged to be ambulatory if they wished. While women were aware of their options for positioning in labour, they agreed to avoid the hands-and-knees position, or positions that approximate it, such as kneeling in bed and using the head of the bed for support.

#### Hands-and-knees

Immediately after randomization, the woman was assisted into the position and asked to maintain it for a total of at least 15 min within a one-hour period. The woman was told she was free to break the 15 min into shorter periods, but to aim for at least 15 min in total during the first hour. Offering the position in this manner allowed for interruptions for care in the labour setting (e.g. changes of patient gown, recording of vital signs) as well as position changes. Her support person(s) were taught how to assist her into the position. Paper “clocks” were provided to assist them with estimating the amount of time spent in hands-and-knees. The nurse demonstrated various ways to get into the position in the hospital bed and on the floor, using a birthing ball, pillows, or the head of the bed for upper body support. On an hourly basis, at the time of the nurse’s regular visit to the labour room, the woman was asked to consider trying hands-and-knees and assisted into it as needed. She was encouraged to maintain the position for as long as it was comfortable. If the woman found it to be uncomfortable, she was free to get out of the position but encouraged to try it again later in labour. A support person or nurse had to be in attendance while the woman was in the position. Hands-and-knees was to be discontinued if it had a negative impact on the fetal heart rate pattern or maternal blood pressure. Position for delivery was determined by the woman and her care providers and was not dictated by the trial protocol.

### Measures

#### Measuring compliance

Prior to the trial onset, we operationally defined compliance with positioning as follows: Women in the hands-and-knees group would try the position at least three times during the hourly intervals between randomization and delivery, and women in the usual care group would not try hands-and-knees position. At hourly visits, a nurse checked the paper “clocks” and inquired if the participant had used the hands-and-knees position during the previous hour.

#### Persistent back pain

At trial entry and on an hourly basis, each participant was asked to rate her level of persistent back pain on a numeric rating scale, ranging from 0 (“no pain”) to 10 (“worst pain imaginable”), and to indicate whether, compared to one hour ago, the persistent back pain was a lot better, a little better, about the same, a little worse, or much worse.

#### Participants’ views

After delivery, each participant was asked to complete a self-administered questionnaire about her experiences. Questionnaire items had been developed and tested in previous trials (Hodnett et al., 2002; Hodnett et al., 2008), and included items which compared their expectations to their experiences, and their willingness to participate in the trial if they had it to do over. Those in the hands-and-knees group were also asked to rate the perceived helpfulness of the position.

Data about labour, birth, neonatal, and postpartum outcomes were obtained by the hospital research nurses from the participants’ medical records.

#### Data analyses

Because it was a small pilot trial and not powered to detect differences in outcomes, results were analyzed descriptively.

## Results

**Figure 1:**
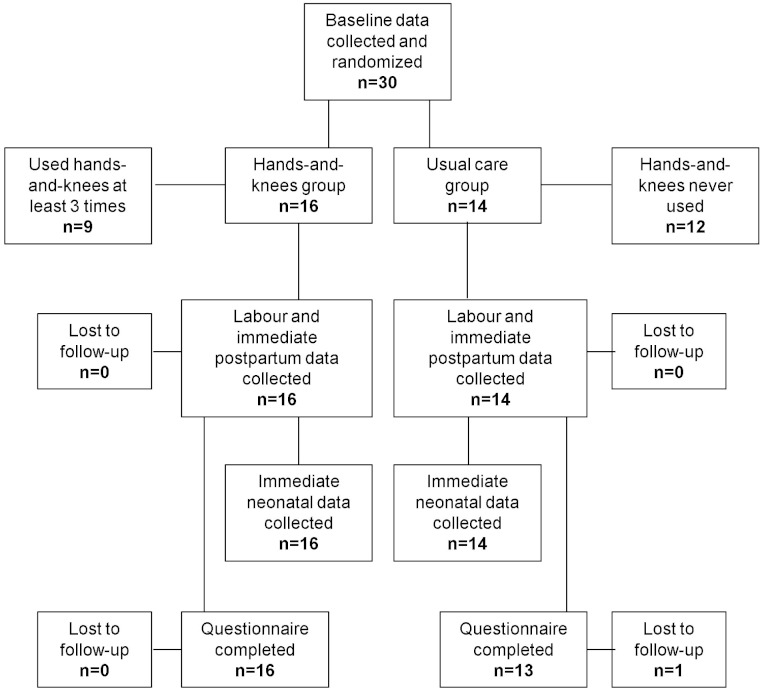
Trial Schema.

Recruitment at the Toronto hospital began in October 2010 and ended in February 2011 after only 11 women were enrolled. Recruitment at the Texas hospital began in October 2011 and ended February 2012, when only 19 women had been enrolled, for a total of 30 women, rather than the desired sample size of 60. Figure 1 shows the flow of participants through the trial. Sixteen women were randomly allocated to the hands-and-knees group and 14 to the usual care group.

### Baseline data

Baseline data are presented in Table 1. Labour onset was induced for 16 women, all of whom were receiving intravenous oxytocin on trial entry. Eighteen had ruptured membranes. Five had epidural analgesia in situ on trial entry. Cervical status was unknown for 11 women. Of the remaining 19, 15 were less than 4 cm dilated upon enrolment (9 in the hands-and-knees group and 6 in the usual care group).

**Table 1:** Baseline characteristics of the sample.

Characteristic	Hands-and-knees	Usual care
	*n* = 16	*n* = 14
Age (Median, IQR)	31 (26.5, 31.0)	25.0 (21.0, 27.0)
Education		
Post-secondary	10	5
High school diploma	4	7
< High school	1	1
Married/stable relationship	15	13
Ethnicity		
Caucasian	12	8
Asian	1	2
African-American/Canadian	1	2
Multiracial	2	2
Attended childbirth education classes	16	14
Support person present	15	13
Spontaneous onset of labour	6	8
Ruptured membranes	11	7
Cervical dilatation within 1 h of entry		
< 4 cm	9	6
≥ 4 cm	3	1
unknown	4	7
Epidural analgesia in situ	3	2
Intravenous oxytocin infusion	11	5
Median (IQR) persistent back pain score	0 (0, 3)	3 (0, 5)

### Compliance

Hands-and-knees position was used 3 or more times by 9 of the 16 women in the hands-and-knees group (Table 2). Two in the usual care group reported they used hands and knees position once during labour.

**Table 2:** Compliance: frequency of usage of hands-and-knees position.

Number of times used	Hands-and-knees	Usual Care
	*n* = 16	*n* = 14
None	3	12
1	1	2
2	3	0
>2	9	0

When asked the reason(s) for the time they spent in hands and knees, a variety of responses were provided. Two women in the hands-and-knees group reported that the hands and knees position was uncomfortable, 1 woman reported experiencing a problem for herself or her baby, and 4 reported other problems which made it impossible to use the position. Three women in the hands-and-knees group and 1 in the usual care group reported that the position was comfortable or decreased their pain, 2 women in the hands-and-knees group and 1 in the usual care group believed their labour progressed better when they were in the position. Six women in the hands-and-knees group reported the reason for the amount of time they used hands-and-knees was that their caregiver suggested they do so. None reported being advised not to use it. Only 2 women, both in the hands-and-knees group, used hands and knees while pushing in second stage labour.

#### Other positions

When asked about other positions used during labour, 9 women in each group reported sitting, while 4 in the hands-and-knees and 6 in the usual care group reported standing/walking. Twenty-three women (11 hands-and-knees position and 12 usual care) reported side-lying, and 9 in the hands-and-knees group and 11 in the usual care group reported lying flat on their backs.

#### Persistent back pain

A total of 299 hourly ratings of persistent back pain were obtained from the 30 participants. No discernable pattern was evident in participants’ hourly ratings of persistent back pain or its intensity relative to the previous hourly rating. Ratings were highly variable, covering the full range of possible scores. Given the low compliance in the hands-and-knees group, it was not possible to explore relationships between use of the position and persistent back pain scores.

### Labour and birth outcomes

Labour and birth outcomes are presented in Table 3. All but 4 women (2 in each group) had intravenous oxytocin (for either induction or augmentation) during first and/or second stage labour. Twenty-seven of the 30 women had regional analgesia, and no woman laboured or gave birth without some form of pharmacologic analgesia. Mean length of time from randomization to delivery was 12.34 h (SD = 6.40) in the hands-and-knees group and 9.65 (SD = 5.62) in the usual care group. Eleven of the 16 women in the hands-and-knees group and 12 of the 14 women in the usual care group had spontaneous vaginal births.

**Table 3:** Labour and birth outcomes.

Outcome	Hands-and-knees	Usual care
	*n* = 16	*n* = 14
Intravenous oxytocin during labour	14	12
Intrapartum analgesia/anesthesia		
None	0	0
Regional analgesia	15	12
Intramuscular opioids	2	2
Dislodged epidural/spinal catheter	3	1
Fell when using hands-and-knees position	0	0
Mean (SD) labour length from randomization to delivery	12.34 (6.40)	9.65 (5.62)
Method of delivery		
Spontaneous vaginal	11	12
Vacuum extraction	1	2
Caesarean	4	0
Perineal trauma	9	9
3rd or 4th degree laceration	(0)	(2)
Postpartum hemorrhage or blood transfusion	0	0
1-Minute Apgar Score <7	2	2
5-Minute Apgar Score <7	0	0
Neonatal respiratory problems	2	3
Transfer to Neonatal Intensive Care Unit	0	2
Median (IQR) length of postpartum hospital stay	47.48 (36.41, 60.05)	47.19 (36.45, 54.62)
Median (IQR) length of neonatal hospital stay	51.18 (36.41, 60.05)	48.48 (36.45, 56.77)

Neonatal outcomes are presented in Table 3. All babies were vertex and alive at birth. No babies had life threatening congenital anomalies, none had major birth trauma, and there were no deaths.

### Participants’ evaluations

All 16 women in the hands-and-knees group and 13 of the 14 in the usual care group completed questionnaires evaluating their labour and birth experiences (Table 4). When asked to compare their experiences with their expectations, responses in both groups were mixed. Responses in both groups were generally positive in regard to intentions to use hands-and-knees position in a future labour. However, when asked how likely they would be to participate in the study again, if they had it to do over, the hands-and-knees group’s responses were more mixed. When asked to rate their overall satisfaction with their birth experiences on a scale of 1–10, ratings ranged from 5 to 10. The hands-and-knees group’s ratings tended to be lower than those in the usual care group. For example, 5 in the hands-and-knees group and none in the usual care group rated their experience as a “5”, while 7 in the hands-and-knees group and 10 in the usual care group rated their experience as a “10”.

**Table 4:** Women’s views of their experiences.

Question	Hands-and-knees	Usual care
	*n* = 16	*n* = 13^a^
My childbirth experience was…		
Much worse or somewhat worse than I expected	8	3
Both better and worse than I expected	3	7
About what I expected	1	1
Much better than I expected	4	2
Plans to try hands-and-knees in a future labour		
Definitely/probably not	3	0
Unsure	2	5
Definitely/probably yes	11	8
Willingness to participate in the study if time went backwards and I had it to do over again		
Definitely/probably not	6	0
Unsure	0	3
Definitely/probably yes	10	10

**Notes.**

a 1 woman did not complete a questionnaire.

## Discussion

The low recruitment rate was unexpected. We selected two large hospitals in which the nursing staff seemed to be very enthusiastic about the trial. One was local, permitting us to have close, ongoing contact, and one was the most successful recruiter in a previous multi-centre trial. However, despite the use of all of the measures which had demonstrated success in our previous large, multi-centre trials, recruitment of participants was extremely slow and fell well below the targets.

Compliance was sub-optimum. Although mean length of time from randomization to delivery was over 12 h, and according to the study protocol hands-and-knees position was to be tried hourly, only 9 participants in the hands-and-knees group achieved our pre-set level of compliance, i.e. at least three attempts in the position. Two in the usual care group violated the protocol and used hands-and-knees position. More than one factor probably contributed to the low compliance. There were constraints on participants’ mobility during labour, as evidenced by the high rates of epidural analgesia, intravenous infusions for oxytocin or other reasons, and routine continuous electronic fetal monitoring. Although women were shown how to achieve hands-and-knees position in bed and under these conditions, doing so took levels of energy and commitment which they (and their care providers) may not have had. In contrast to the current pilot trial, in our previous trial of hands-and-knees position, women had an identified problem – a fetus in the OP position – which served as a motivator for trying hands-and-knees position as a potential solution to the problem. Furthermore in our LPT1 trial, women were only asked to use hands-and-knees position within the first hour after randomization, and thus the time and energy commitment was much less than in the present trial.

While the purpose was not to detect differences in labour and birth outcomes, and the pilot trial was far too small to do so, there was no evidence of benefit on birth outcomes, from a policy of encouraging frequent use of hands-and-knees position during labour.

## Conclusion

Because of the very slow enrolment rates, poor compliance in the hands-and-knees group, mixed responses about the perceived helpfulness of the position, and lack of suggestion of a beneficial effect on birth outcomes, we concluded that a definitive trial of hands-and-knees was not feasible, e.g. could not be justified in terms of the time and expense required to conduct it, in hospitals with similar characteristics to the ones in our current and prior (Hodnett et al., 2002; Hodnett et al., 2008) labour trials. However a definitive trial of repeated hands-and-knees positioning may be feasible and desirable, with modifications to the eligibility criteria and careful selection of settings. For example, the problems of poor compliance and lack of perceived helpfulness may be overcome by enrolling only women with a defined problem, such as suspected OP position or persistent back pain. In addition, the problem of poor compliance may be lessened in settings in which non-recumbent positions are common during labour.

## Supplemental Information

10.7717/peerj.25/supp-1 Supplemental Information 1 CONSORT Checklist

10.7717/peerj.25/supp-2 Supplemental Information 2 Trial Protocol

10.7717/peerj.25/supp-3 Supplemental Information 3 Consent Form

10.7717/peerj.25/supp-4 Supplemental Information 4 Trial Schema

## Additional Information and Declarations

### Clinical Trial Registration

The following information was supplied regarding Clinical Trial registration: www.clinicaltrials.gov. Registration Number: NCT01720004.
