# Supplementary material for: Repeated hands-and-knees positioning during labour: a randomized pilot study
Source: PeerJ. 2013 Feb 12;1:e25. doi: 10.7717/peerj.25 (PMC3629039; doi:10.7717/peerj.25)
Supplement: Supplemental Information 2 [file peerj-01-25-s002.pdf]

Protocol Registration Receipt
10/30/2012

A Pilot Randomized Controlled Trial of Repeated Hands-and-Knees Positioning During Labour (LPT2)

This study has been completed.

Sponsor:	University of Toronto
Collaborators:	Canadian Institutes of Health Research (CIHR)
Information provided by (Responsible Party):	Ellen Hodnett, University of Toronto
ClinicalTrials.gov Identifier:	NCT01720004

Purpose

The investigators designed a pilot randomized controlled trial to assess the feasibility and acceptability of repeated hands-and-knees positioning during labour. The objectives were 1) to provide an estimate of enrollment rates, 2) to assess compliance with the study protocol by participants and care providers, 3) to obtain women's views about their experiences using the hands-and-knees position, and 4) to provide estimates of treatment effects to inform the sample size calculation for a large trial.

Condition	Intervention	Phase
Pregnancy, Childbirth and the Puerperium	Repeated hands-and-knees positioning during labour	Phase 1

Study Type: Interventional
Study Design: Treatment, Parallel Assignment, Single Blind (Investigator), Randomized, N/A
Official Title: Repeated Hands-and-Knees Positioning During Labour: A Pilot Randomized Controlled Trial

Further study details as provided by Ellen Hodnett, University of Toronto:

Primary Outcome Measure:

- Compliance [Time Frame: from randomization to delivery] [Designated as safety issue: No]
  Use of hands-and-knees position for at least 15 minutes hourly during hospital labour.

Secondary Outcome Measures:

- Persistent back pain [Time Frame: hourly during labour, from randomization to delivery] [Designated as safety issue: No]
  Persistent back pain intensity rating measured hourly during hospital labour.
- women's views [Time Frame: assessed prior to hospital discharge] [Designated as safety issue: No]
  women's views of their birth experiences, including satisfaction with care and care providers, views about hands-and-knees positioning, willingness to use hands-and-knees position in a subsequent labour, comparison of expectations versus experiences of labour. The measures used to assess women's views had been developed for and used in prior trials of forms of intrapartum care by Hodnett and colleagues. Most questions were Likert scales or categorical items.

Other Pre-specified Outcome Measures:

- Method of birth [Time Frame: at delivery] [Designated as safety issue: No]
  Spontaneous vaginal, assisted vaginal (vacuum or forceps), Caesarean
- pharmacologic analgesia [Time Frame: Initiated during labour] [Designated as safety issue: No]
  regional analgesia or intramuscular analgesia administered during first or second stage labour
- oxytocin during first or second stage labour [Time Frame: from randomization until end of second stage] [Designated as safety issue: No]
  any oxytocin infusion
- dislodged epidural catheter [Time Frame: from randomization until delivery] [Designated as safety issue: Yes]
  dislodged epidural catheter
- Fall [Time Frame: from randomization until delivery] [Designated as safety issue: Yes]
  Mother fell while attempting hands-and-knees position
- perineal trauma [Time Frame: at delivery] [Designated as safety issue: No]
  any perineal trauma (episiotomy and/or laceration0 requiring suturing
- maternal postpartum complications [Time Frame: between delivery and hospital discharge] [Designated as safety issue: No]
  postpartum hemorrhage or complication requiring prolonged stay
- Apgar Score [Time Frame: at one and five minutes after birth] [Designated as safety issue: No]
  Neonatal Apgar Score
- length of hospital stay [Time Frame: from delivery to discharge] [Designated as safety issue: No]
  length of stay for mother and baby after birth
- admission to neonatal intensive care unit [Time Frame: between birth and hospital discharge] [Designated as safety issue: No]
  newborn admitted to neonatal intensive care unit
- labour length [Time Frame: from randomization until delivery] [Designated as safety issue: No]

Length of time between randomization and delivery

Enrollment: 30
Study Start Date: October 2010
Study Completion Date: February 2012
Primary Completion Date: February 2012

Arms	Assigned Interventions
Experimental: Repeated Use of Hands-and-Knees<br>The intervention was repeated use of hands-and-knees position during labour. Participants were asked to try it for at least 15 minutes every hour, from randomization until delivery. They were not required to use it for delivery.	Repeated hands-and-knees positioning during labour<br>Details are in the Arm Description.
No Intervention: Usual care<br>Participants were asked to refrain from using hands-and-knees position at any time from randomization to delivery. They were free to use any other position.	

Women were enrolled in the pilot randomized controlled trial at two hospitals, one in Canada and one in the USA. Nurses at both hospitals were trained in how to assist women into the hands-and-knees position in bed. Repeated hands-and-knees position was defined as attempts to use the position for 15 minutes, hourly from randomization until delivery. Women were not asked to assume hands-and-knees for delivery.

Eligibility

Genders Eligible for Study: Female
Accepts healthy volunteers.

Inclusion Criteria:

- nulliparous;
- >37 weeks 0 days gestation
- in established early labour
- anticipating a vaginal delivery of a single fetus in the cephalic position
- competent to give informed consent .

Exclusion Criteria:

- delivery was anticipated within 3 hours

- a medical contraindication or physical limitation such that hands-and- knees position was contraindicated
- had a doula or midwife who encouraged the use of hands-and-knees position.

Contacts and Locations

Locations

United States, Texas

Texas Health Harris Methodist Hospital
Fort Worth, Texas, United States, 76104

Canada, Ontario

Toronto East General Hospital
Toronto, Ontario, Canada, M4C 3E7

More Information

Responsible Party:	Ellen Hodnett, Professor, University of Toronto
Study ID Numbers:	IGO-103690
Health Authority:	Canada: Canadian Institutes of Health Research
