# Supplementary material for: Repeated hands-and-knees positioning during labour: a randomized pilot study
Source: PeerJ. 2013 Feb 12;1:e25. doi: 10.7717/peerj.25 (PMC3629039; doi:10.7717/peerj.25)
Supplement: Supplemental Information 3 [file peerj-01-25-s003.docx]

**Consent Form**

**Labour Position 2 Pilot Study**

**Principal Investigator: (Name)**

The best position for a baby during labour and birth is with the head down, and the back of the head turned out towards the mother’s abdomen. If the back of the baby’s head is toward the mother’s back, the mother may feel a constant back pain, and labour may be slower. The baby’s head may rotate (move from the best position to other positions) several times during labour. Approximately 5% of babies do not turn to the best position before delivery, and in such cases there is a greater chance that the baby will be delivered by forceps, vacuum extraction, or caesarean.

In Canada, women who are in labour in hospitals most often lie on their side or are in a semi-sitting position. While some doctors and nurses believe these positions, especially side-lying, will help the baby’s head to rotate to the best position, others feel that hands-and-knees position may help by using gravity to rotate the baby’s head.

Hands-and-knee position involves the labouring woman “on all fours,” i.e. like a baby who is crawling, so that her abdomen is suspended and her hips are at right angles to the floor or bed. The hands-and-knees position can be used by women who have had low dose epidural analgesia, and by those who are connected to electronic fetal monitors and IVs.


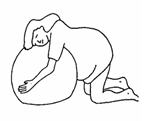

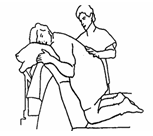

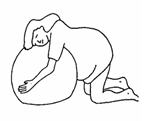

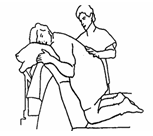


Variations on hands-and-knees position

Illustration by Sharma de la Cruz © Ruth Archeta. Reprinted by permission from Penny Simkin & Ruth

Archeta (*Labour Progress Handbook* Blackwell Science 1999)

There is little research on the effects of any type of position during labour, so we do not know what the actual effects of repeated use of the hands-and-knees position during labour will be for women, their babies, and for the process of labour and birth. Thus we have little basis for advice to women about positions they may find helpful.

**Purpose of Study:**

The Labour Position 2 Study (LPT2) will compare the use of the ‘usual’ positions during labour and the repeated use of hands-and-knees position with the use of only the ‘usual’ positions during labour. The study is a randomized trial. Randomization means that neither the study participants nor their doctors or nurses choose whether the labouring mother uses hands-and-knees position or not. Instead this choice is made randomly, in a process similar to a coin flip, using a computer. This is the best study design for determining if using hands-and-knees position affects the process of labour and birth and how women feel about it afterwards. Before we design a big trial that will involve several hospitals and thousands of women, we need to conduct a small pilot study of 60 women, to find out how women feel about using the hands-and-knees position. Because you are in labour with your first baby, you are invited to participate in the LPT2 Pilot Study. The trial is headed by Dr. Ellen Hodnett, Professor of Nursing in the University of Toronto and funded by the Canadian Institutes of Health Research (Grant # IGO-103690) and taking place only at Toronto East General Hospital.

**Description of Study Groups:**

Group #1: Usual Care. If you are allocated to the usual care group, during labour you may use a variety of positions, including sitting, lying on your side. semi-sitting in bed, or walking. You will be asked not to use the hands-and-knees position if at all possible, unless your nurse or doctor feels it is very important that you do so. At the end of each hour you are in labour, you (or your labour support person) will be asked to mark down which labour positions you have been using. This should take 1 minute or less each time.

Group #2: Hands-and-Knees. If you are allocated to the hands and knees group, during labour you may use all of the ‘usual’ positions (e.g. walking, sitting, lying on your side or semi-sitting in bed) AND you will use the hands-and-knees position. Right after you join the study, a nurse will help you get into the hands-and-knees position and show you various ways to get into the position in the hospital bed and on the floor, using a birthing ball, pillows, or the head of the bed for upper body support (see pictures above for some examples). Your labour support person will be shown how to help you. You will be asked to use the hands-and-knees position for at least 15 minutes during the first hour after you join the study. For the rest of labour you will be encouraged to do the same -- use the hands-and-knees position for at least 15 minutes of every hour. You do not have to do the 15 minutes all at once; you can break the time up into shorter periods if you wish. You will never be alone while you are using the hands-and-knees position, your support person or a nurse will be with you. You will be given a paper ‘clock’ so that you or your support person can mark down when you use the hands-and-knees position each hour. This should take just a minute or two to complete each hour. If you find the hands-and-knees position uncomfortable you will be free to stop using it at any time. Your nurse may encourage you to re-try the position later in labour, as things change over time and you may find it comfortable once again. However you will never have to use the hands-and-knees position if you do not want to. If at any time your nurse or doctor think that the hands-and-knees position is causing problems for you or your baby, they will ask you to stop using it. The position you are in for the actual birth of the baby will be a decision between you and your doctor.

This study will not affect your decision to have an epidural. If you have an epidural in place now (before joining the study) you are still able to participate. It is safe for you to use the hands-and-knees position with an epidural. If you do not have an epidural at this time, this study will not affect your decision later on. This decision would be made with your doctor and nurse as usual. You would still be in the study and could continue to use the hands-and-knees position after you have your epidural.

For both groups: Right after you join the study and every hour during labour you will be asked to rate how much back pain you are feeling. You will be given a scale to help with this. This should take 1 minute or less each time. Before you go home from the hospital after having your baby you will be asked to complete a questionnaire about your childbirth experience and your participation in the study. It will take 5-10 minutes to complete this questionnaire. After you have given birth, a research assistant will collect information from your hospital record, about medical aspects of your labour, birth, and your baby’s health.

In all other respects the nursing and medical care you and your baby receive will not be different because you participate in the LPT2 Pilot Study.

**Risks and Benefits:**

Some women may find that their wrists or knees get tired when using the hands-and-knees position. You can take rest breaks and use another position if this happens. In a previous small study there was one episode of a fetal heart rate deceleration that occurred during the use of hands-and-knees. The woman was assisted out of the position and the fetal heart rate pattern returned to normal immediately. No other complications have been reported from the use of hands-and-knees. Fetal heart rate decelerations can also occur when using the “usual” positions during labour. In such cases changing positions often corrects the problem.

It is possible that your blood pressure could be affected by using hands-and-knees or other positions during labour, but it seems reasonable to assume that getting out the position would correct this as was noted above for the baby’s heart rate. Other possible but very rare risks are that the epidural catheter could become dislodged when moving in and out of the hands-and-knees position, or you might lose your balance and fall over from the position. To minimize these risks you will not be alone when using hands-and-knees; you will have your support person or a nurse there to help you.

A benefit of the study is the opportunity to tell others of your labour and delivery experiences. Other studies have found that women appreciate an opportunity to tell researchers their feelings about their childbirth experiences. The results of the study will help to decide on the best positions for women like you to use during labour.

**Alternative Treatments:**

If you choose not to participate, you will receive the usual nursing care during labour without any change in your or your family’s present or future care. If you do join the study you are free to withdraw at any time without explanation and without any change in your or your family’s present or future care.

**Confidentiality:**

All information about you and your baby will be kept strictly confidential. A code number, rather than your name, will be used on all study forms and questionnaires. The signed consent forms will be kept in separate, locked cabinets, accessible only to research staff. No information will be released or printed that would disclose the identity of any of the participants. If you wish, at the end of the study a summary of the results will be made available to you.

**Questions regarding participation and contact information:**

If you have questions about the study please contact:

(Name of Site Investigator) Tel: xxxxxxx or

(Name of Principal Investigator) Tel: xxxxxxxx

If you have concerns or questions about your rights as a research participant in the study you may contact (Name), Chair of the XX Hospital Research Ethics Board at xxxxx.

**Labour Position 2 Pilot Study**

**Consent:**

I have been able to discuss this study and all my questions have been answered. I consent to take part in the study with the understanding I may withdraw at any time without affecting my care.

By signing the present form, I expressly consent to the collection and use of my personal data in accordance with this document.

**SIGNATURES**

1. I carefully read the information in this Consent Form. I received an explanation of the nature, purpose, duration and possible effects of the trial. The possible risks and benefits of the trial and the other treatments available have been explained to me. I was given time and opportunity to ask questions about the study. All my questions were answered to my satisfaction.
2. I voluntarily consent to take part in this research study.
3. I have been informed about the compensation and/or treatment available to me in the event of study-related injury.
4. I will be given a copy of this signed and dated Consent Form.

___________________________ ____________________________ ____________

# Subject’s Name (Print) Subject’s Signature Date

I confirm that I have explained the nature, purpose and foreseeable effects of the trial to the patient whose name is printed above. The patient consented to take part in this study by signing and dating this form.

___________________________ ____________________________ ____________

# Name (Print) Signature Date
