# Supplementary material for: Repeated hands-and-knees positioning during labour: a randomized pilot study
Source: PeerJ. 2013 Feb 12;1:e25. doi: 10.7717/peerj.25 (PMC3629039; doi:10.7717/peerj.25)
Supplement: Supplemental Information 4 [file peerj-01-25-s004.docx]

Figure 2. Trial Schema

Baseline data collected and

randomized

**n=30**

Hands-and-Knees Group

**n=16**

**n=16**

**n=16**

Lost to

follow

-

up:

**0**

Lost to

follow

-

up:

**0**

Labor and immediate

postpartum data collected

**n=**

**16**

Labor and immediate

postpartum data collected

**n= 14**

Immediate

neonatal data

collected

**n=**

**16**

Questionnaire completed

**n=**

**16**

Questionnaire completed

**n=**

**13**

Immediate

neonatal data

collected

**n=**

**14**

-

Used hands-and-knees at least 3 times

**n=9**

Usual Care Group

**n=14**

Hands-and-knees never used

**n=12**
